# Variation characteristics and the impact of urbanization of extreme precipitation in Shanghai

**DOI:** 10.1038/s41598-022-22352-4

**Published:** 2022-10-21

**Authors:** Yali Mou, Xichao Gao, Zhiyong Yang, Tianyin Xu, Jie Feng

**Affiliations:** 1 China Institute of Water Resources and Hydropower Research, Beijing, 100038 China; 2 State Key Laboratory of Simulation and Regulation of Water Cycle in River Basin, China Institute of Water Resources and Hydropower Research, Beijing, 100038 China

**Keywords:** Hydrology, Natural hazards, Climate change

## Abstract

With the rapid development of urbanization, the characteristics of extreme precipitation in urban areas have changed significantly. Revealing the spatial and temporal distribution of extreme precipitation under changing environment is the basis of scientific response to the urban flood. Trends of extreme precipitation at 95% and 99% thresholds in Shanghai and the influence of urbanization on them were analyzed. The results show that: (1) The precipitation threshold limit value for each site are 5.7 ~ 6.3 mm at 95% with a variation factor of 0.04, and 14.3 ~ 17.16 mm at 99% with a variation factor of 0.06. The precipitation thresholds under 99% conditions were more significantly different among stations. (2) The extreme precipitation at each site has been increasing over the past 50 years, and the growth rates of 95% and 99% extreme precipitation are 8.02~11.46%/10a and 7.11~16.86%/10a, respectively. The growth rate of extreme precipitation is significantly higher than that of average precipitation, while the extreme part of the precipitation probability distribution increases considerably. There is a strong variability in extreme precipitation in this region, while the 99% threshold precipitation varies more. (3) The extreme precipitation in Shanghai is significantly positively correlated with the urbanization of the area around the site. Urbanization has an increasing effect on regional extreme precipitation, with more extreme precipitation and greater growth rate in highly urbanized areas.

## Introduction

The regional and even global precipitation structure has changed significantly under global warming^1–4^. The increasing impact of human activities on the global and regional water cycle is becoming more and more significant, leading to precipitation structure in regions with high human activities changing more obviously^5, 6^. Climate change and rapid urbanization have also significantly altered extreme precipitation characteristics in urbanized areas. The extreme precipitation is the main reason for urban flooding. Trends of its frequency, as well as spatial and temporal distribution patterns under the changing environment, have attracted attention of many researchers^7–11^. Donat et al.^12^ noted a clear trend of increasing extreme precipitation globally in the twenty-first century. Gu et al.^13^ pointed out that the occurrence of extreme precipitation on an interannual scale in China is largely dependent on the variability of climate indicators and exhibits non-stationary characteristics. The regionality and persistence of extreme precipitation are generally higher in southern China, while those in northern areas are quite low^14, 15^. Relevant studies on the Three River Plain, the Pearl River Basin, the Yangtze River Delta, the Huaihe River Basin, etc. demonstrated that extreme precipitation in different regions shows different trends, and its spatial distribution within the region is also different^16–19^.

Many studies have been done on extreme precipitation events, including the effects of urbanization on the extreme precipitation. Ren et al.^20, 21^ analyzed the trends and causes of spatio-temporal variation of precipitation in Chinese mainland indicating an increasing frequency and intensity of extreme intense precipitation events, and a tendency toward shorter duration for single extreme intense precipitation events. It was pointed out that the main source of the systematic deviation of modern climate is that urbanization process increases the frequency of extreme precipitation events and the accumulated rainfall. Tan et al.^22^ analyzed the changes in the spatial and temporal patterns of heavy rainfall in different urbanized regions of China and found that the interannual and interdecadal heavy rainfall amounts, days of rainfall and rainfall intensity in the Beijing-Tianjin-Hebei region show a decreasing trend, while that in the Yangtze River Delta and Pearl River Delta regions show an increasing trend. Liang et al.^23^ studied the relationship between urbanization rate and spatial distribution of precipitation in Shanghai. Results showed that spatial difference increases with rapid acceleration of urbanization. Spatial distributions of annual rainfall and rainstorm frequency exhibits distinct urban “rain-island” feature during rapid acceleration period of urbanization while the case is opposite during slow acceleration period. Miao et al.^24^ calculated 10 extreme precipitation indices in the Beijing-Tianjin-Hebei region based on daily precipitation data from 25 meteorological stations from 1961 to 2017 and found the same results with Tan et al. Song et al.^25^ analyzed the spatial and temporal evolution of extreme precipitation in Beijing and compared the differences in three extreme precipitation indices (frequency, precipitation amount, and contribution ratio) between urban and suburban areas in two phases (1960–1985 and 1986–2012), showing that the daily extreme precipitation in Beijing presented a significant decreasing trend, while the three extreme precipitation indices were higher in urban areas in the latter phase. Yang et al.^26, 27^ also conducted a study on the impact of urbanization on short-duration heavy precipitation in Beijing based on hourly precipitation data, showing that short-duration heavy rainfall in Beijing is strongly correlated with urbanization, and the urban heat island effect is more obvious in summer. Huang et al.^28^ used spatial analysis, linear regression, and trend test to analyze the extreme rainfall in the highly urbanized areas of the Pearl River Delta, finding a significant increase, while no significant change is witnessed in other adjacent areas. The storm rainfall pattern in the Pearl River Delta is dominated by unimodal patterns, which easily leads to an increase in rainstorm flooding events. There are also studies on metropolitan areas in China that similarly show that extreme precipitation occurs more frequently and with greater intensity in urban areas^29, 30^. Most studies showed that urbanization has an enhanced effect on extreme precipitation events, but this is not the case in all regions. The physical mechanism of the impact of urbanization on precipitation is complex, and the impact itself is not consistent. There is some spatial variability in the effects of urbanization on extreme precipitation: Zhao et al.^31^ observed that urbanization in the Beijing-Tianjin-Hebei region has no significant effect on extreme precipitation changes; He et al.^32^ pointed that urbanization increases extreme precipitation magnitude and days of precipitation in urban areas but reduce extreme precipitation days in peri-urban areas, which means that the urbanization can affect the extreme precipitation. From the literature, we can learn that urbanization has significant effects on extreme precipitation. However, at least to our best knowledge, the influence of urbanization degree on the change rate of extreme precipitation has not been studied. In this paper, trends of the extreme precipitation and its correlation with urbanization degree in Shanghai has been investigated. The results can deepen our understanding of the influence of urbanization on extreme precipitation in urban areas and provide support for urban flood control.

## Materials and methods

### Study area

**Figure 1:**
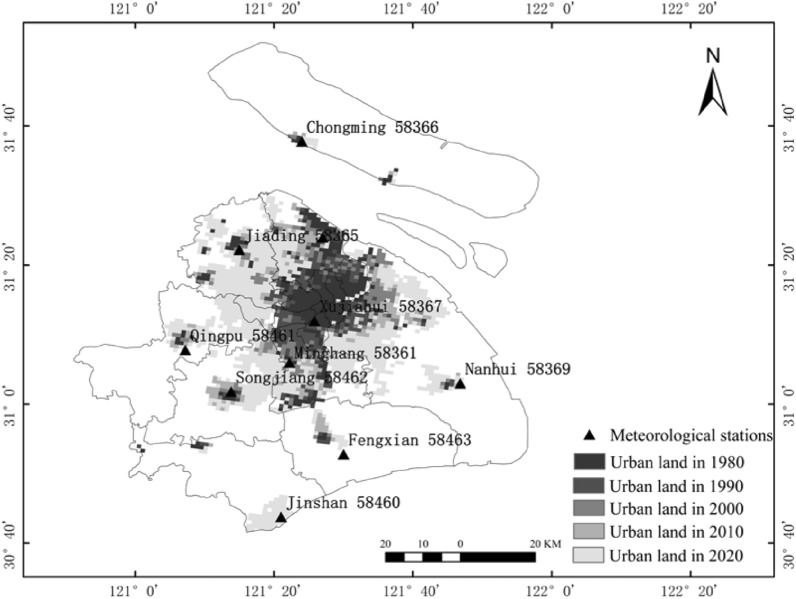
Study areas and meteorological stations.

Shanghai, a highly urbanized area in China, is located on the eastern edge of the Asian continent, between 120°51' ~ 122°12'E, 30°40' ~ 31°53'N (Fig. 1). It is in an estuarine deltaic alluvial plain with a low topography and an average elevation of about 4 m above sea level. Shanghai has a subtropical monsoon climate with simultaneous rain and heat, abundant rainfall, and four distinct seasons. The annual average temperature of Shanghai is about 16 °C and the annual rainfall is 1100 mm. Heavy precipitation is very frequent^33^. As one of the mega-cities in China, Shanghai has a rapid urbanization development and high urbanization rate. In 2020, it has a GDP of 3,870,058 million yuan, a resident population of 24,883,600, and an urbanization rate of 89.30% of the resident population^34^.

### Data

Hourly precipitation data is used in this study, obtained from the National Meteorological Information Centre of the China Meteorological Administration (http://data.cma.cn/). There are 11 meteorological stations within Shanghai under this data. Of these, 1 site began observations in 1997, 2 sites had more missing data before 1978, and the remaining 8 sites had more complete data series. Therefore, considering the availability of the data series, we finally selected 10 stations for the analysis of precipitation in the study area (Table 1). Missing data were within 1% for all stations, and missing data were estimated by linear interpolation for the flood season (April–October) and recorded as 0 mm for the non-flood season (November-March)^25^. Knowing that all 10 stations had been relocated (Table 1)^35^, we examined the homogeneity of the mean hourly precipitation at each station using the commonly used sliding t-test and Mann–Kendall test^36–38^. The test results showed that the relocation of meteorological stations did not affect the homogeneity of the precipitation data series, and this data is credible and can be used as the original data for this paper, as detailed in Annex 1.

**Table 1:** Stations information.

Station number	Station name	Time series	Relocation time^35^
58361	Minhang	1978–2020	1993-11-01
58362	Baoshan	1971–2020	2003-01-01
58365	Jiading	1971–2020	1999-08-13
58366	Chongming	1971–2020	2003-05-01
58367	Xujiahui	1971–2020	1999-07-01
58369	Nanhui	1971–2020	2001-11-01
58460	Jinshan	1971–2020	2003-08-01
58461	Qingpu	1971–2020	1999-01-01
58462	Songjiang	1971–2020	2003-08-01
58463	Fengxian	1972–2020	1997-01-01;2010-01-01

The land use data include five periods of 1980, 1990, 2000, 2010, and 2020, all of which are derived from the spatial distribution data of remote sensing monitoring of land use types nationwide by the Resource and Environment Science and Data Center of the Chinese Academy of Sciences (https://www.resdc.cn/Default.aspx). The data was generated by manual visual interpretation based on Landsat TM imagery from the U.S. Landsat satellite. The land use types include 6 primary types of arable land, forest land, grassland, water, residential land and unused land, and 25 secondary types. The urban land used in this paper is a subtype of residential land.

### Methodology

1. Determination of extreme precipitation. There are three main methods to determine extreme precipitation events^24^: (i) the fixed threshold method; (ii) the standard deviation method; and (iii) the percentile threshold method, among which the percentile threshold method is currently the most common method. It identifies extreme precipitation events by defining a certain percentile value as the threshold for extreme values, which can better reflect the extreme rainfall variability in each region as well as the differences between regions^39^. The urbanization rate among the regions represented by each station in Shanghai is relatively large, there are obvious differences in extreme rainfall between regions. The use of a single absolute threshold cannot reflect the differences in extreme rainfall between regions with different degrees of urbanization. To analyze the differences among the sub-regions in the study area, the percentile threshold method was selected to determine extreme precipitation events in this paper. We selected two different percentile thresholds of 95% and 99%. The 95th and 99th percentile precipitation values in ascending order for hourly precipitation greater than 0.1 mm were used as the threshold for extreme precipitation at that station^28^, and extreme precipitation was considered to occur when hourly precipitation exceeded that extreme precipitation event threshold. In this paper, extreme precipitation is selected as the index for analysis, which refers to the sum of hourly rainfall over the threshold value in a year.
2. Trend and correlation analysis. In this study, the Mann–Kendall trend test, Linear regression, Pearson correlation analysis and Precipitation anomaly percentage were used to analyze the trend characteristics of the time series and the correlation of extreme precipitation and urbanization intensity.

(i) Mann–Kendall trend test. The Mann–Kendall test (MK trend test) is a non-parametric test. The advantage of this method is that it does not require the sample to follow a certain distribution and is not disturbed by a few outliers. It searches for a trend in a series without specifying whether the trend is linear or nonlinear^40^. This method determines the trendiness of the series by the statistical variable $Z$. The significance level $\alpha = 0.05$ and the critical value $U_{0.05} = \pm 1.96$ were generally taken. For $Z > 1.96$ ($Z < - 1.96$), the upward (downward) trend of the series is significant with 0.05 significance level.
(ii) Linear Regression. Linear regression is a type of regression analysis that models the relationship between one or more independent and dependent variables using a least square function called a linear regression equation. The algorithm mainly uses regression analysis in mathematical statistics to determine the degree of relationship between correlated quantities and interrelated dependencies between predicted quantities. It is an approximation to determine the linear relationship of a set of data with the model equation $y = ax + b$. The Regression coefficients ($a$ and $b$) are solved by ordinary least squares.
(iii) Pearson Correlation Analysis. The Pearson correlation coefficient is used to measure the correlation between two variables (linear correlation) with a value between − 1 and 1. T-test was used for hypothesis testing.
(iv) Precipitation anomaly percentage (PAP). PAP is a deviation from the normal condition of annual precipitation as estimated below. The calculation formula is $PAP_{i} = \left( {p_{i} - \overline{p}} \right)/\overline{p }$, $\overline{p} = \mathop \sum \limits_{i}^{n} p_{i }$ . In this study, PAP index was used to calculate the extreme precipitation characteristics. $p_{i }$ is annual extreme precipitation, $\overline{p}$ is long-term mean annual extreme precipitation, and $n$ is the number of data.

## Results

### Extreme precipitation

**Figure 2:**
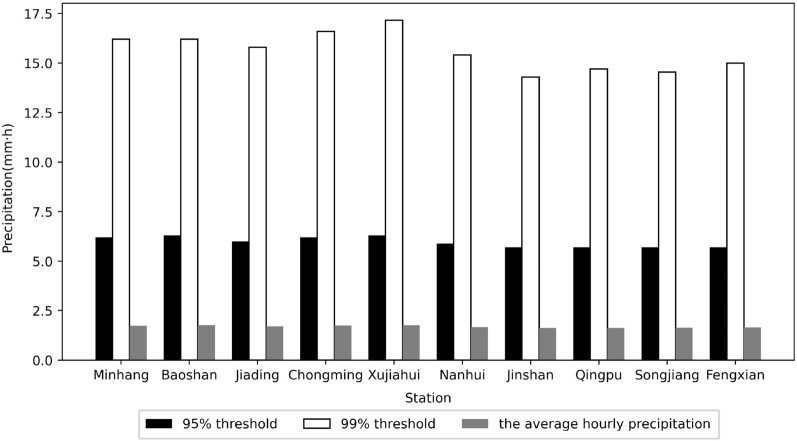
95% and 99% threshold limit value for each station.

We compared the precipitation under three conditions. Two thresholds of 95% and 99% and the average (the average value of hour-by-hour precipitation greater than 0.1 mm at each station) of hourly precipitation data for each station in Shanghai are shown in Fig. 2 and Table 2. Meanwhile, the range and coefficients of variation between stations of the three conditions were compared (Table 3). The average hourly precipitation at each station ranged from 1.62 to 1.77 mm, with a range (R) of 0.15 and a coefficient of variation (CV) of 0.03; 95% of the precipitation threshold limit value varied from 5.7 to 6.3 mm per hour, with a range of 0.6 and a coefficient of variation of 0.04; and 99% of the precipitation threshold limit value varied from 14.3 to 17.16 mm per hour, with a range of 2.86 and a coefficient of variation of 0.06. It can be seen that there are few differences of each site but there are still some differences in terms of comparing the three conditions. The average hourly precipitation differences among stations are the smallest, while the 95% of the precipitation threshold limit value differences are larger, and the 99% of the precipitation threshold limit value differences are the largest. This indicates that the more extreme precipitation shows more significant differences between stations.

**Table 2:** 95% and 99% threshold limit value for each station.

Station number	Station name	95% threshold/mm h^−1^	99% threshold/mm h^−1^	The average hourly precipitation/ mm h^−1^
58361	Minhang	6.2	16.2	1.73
58362	Baoshan	6.3	16.2	1.77
58365	Jiading	6.0	15.8	1.71
58366	Chongming	6.2	16.6	1.74
58367	Xujiahui	6.3	17.16	1.76
58369	Nanhui	5.9	15.4	1.66
58460	Jinshan	5.7	14.3	1.62
58461	Qingpu	5.7	14.7	1.62
58462	Songjiang	5.7	14.55	1.63
58463	Fengxian	5.7	15	1.65

**Table 3:** Site differences in extreme precipitation thresholds and mean hourly precipitation.

Indices	95% threshold	99% threshold	The average hourly precipitation
R	0.6	2.86	0.15
CV	0.04	0.06	0.03

### Temporal variation of extreme precipitation

To investigate the temporal variation characteristics of extreme precipitation events in Shanghai, we used MK trend test to perform statistical significance for temporal trend analysis. Table 4 shows the absolute statistics of each station series are greater than 0, indicating an increasing trend of extreme precipitation for all stations in Shanghai from 1971 to 2020. The increasing trend for all sites at the 95% threshold was statistically significant. All sites except Jinshan and Songjiang were statistically significant at the 99% threshold.

**Table 4:** Trend identification results of MK trend test.

Station name	Z	
	95%	99%
Minhang	2.8466*	2.2815*
Baoshan	3.3464*	2.2580*
Jiading	3.4601*	2.7940*
Chongming	2.8915*	2.7940*
Xujiahui	3.0052*	2.5179*
Nanhui	2.6316*	2.9402*
Jinshan	3.1595*	1.2346
Qingpu	2.7453*	2.8915*
Songjiang	2.7940*	1.8844
Fengxian	2.9221*	2.2929*

The absolute statistic of series is compared with the threshold of 1.96 with 0.05 significance level. If being bigger than 1.96, the trend of the analyzed series is statistically significant (denoted as ‘‘*’’); if being smaller than 1.96, the trend of the analyzed series is not statistically significant.

**Figure 3:**
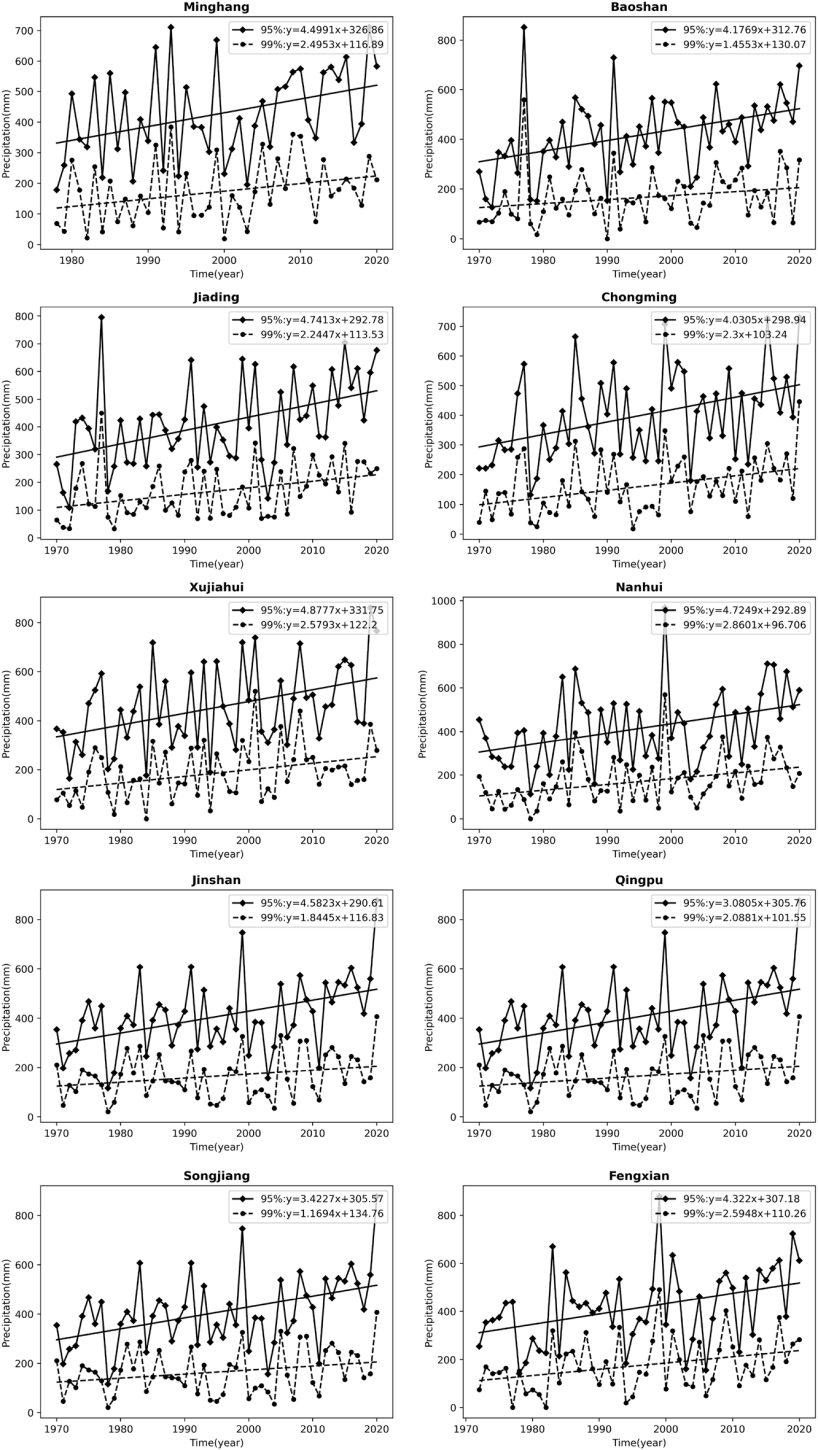
Trends of extreme precipitation under different threshold conditions at each station.

**Figure 4:**
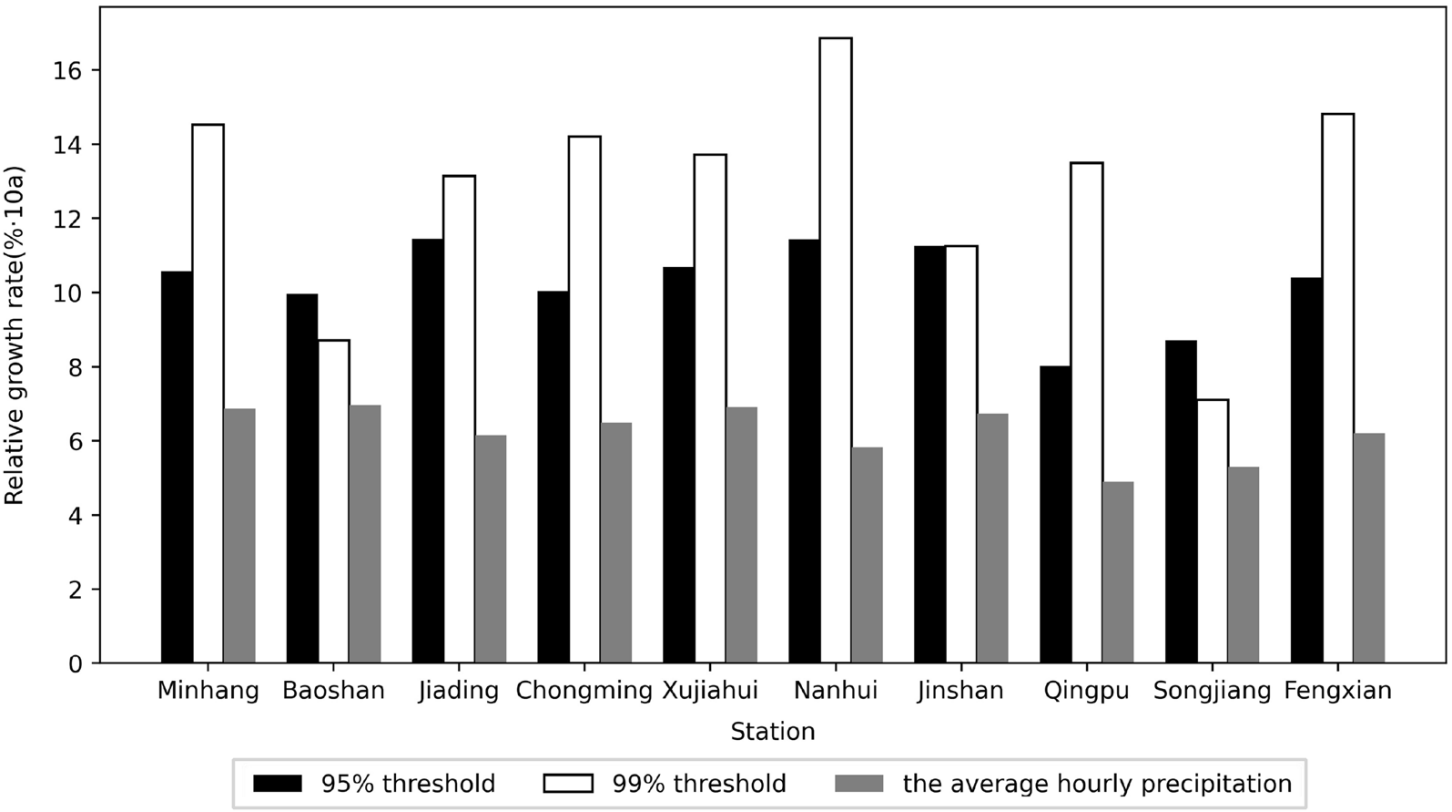
Relative growth rate of extreme precipitation under different threshold conditions at each station.

In addition, a linear regression method was used to analyze the trend of precipitation extremes (Fig. 3). Consistent with the results of the MK trend test, Fig. 3 shows an increasing trend of the extreme precipitation at all stations in Shanghai. Moreover, to exclude the influence of the baseline rainfall threshold on the growth rate, we further calculated the relative growth rate of extreme precipitation at each station, i.e., the relative growth rate of extreme precipitation at each station was obtained by using "absolute growth rate/average extreme precipitation", as shown in Fig. 4 and Table 5. In addition, the relative rate of increase of the average precipitation at each station was also calculated to compare the differences in the changes of extreme precipitation and the average precipitation in the study area. As shown in Fig. 4 and Table 5, the relative growth rate of average precipitation ranges from 4.9 to 6.96%/10a, from 8.02 to 11.46%/10a under 95% threshold conditions, and from 7.11 to 16.86%/10a under 99% threshold conditions. The growth rate of extreme precipitation is significantly higher than that of average precipitation, and most of the stations show a more significant growth rate of extreme precipitation under 99% threshold conditions.

**Table 5:** Relative growth rate of extreme precipitation under different threshold conditions at each station.

Station name	Relative rate of increase of average precipitation/%·10a	95% threshold			99% threshold		
		Absolute growth rate/mm·10a	Average extreme precipitation/mm	Relative growth rate/%·10a	Absolute growth rate/mm·10a	Average extreme precipitation/mm	Relative growth rate/%·10a
Minhang	6.87	44.99	425.84	10.57	24.95	171.79	14.53
Baoshan	6.96	41.77	419.27	9.96	14.55	167.18	8.71
Jiading	6.15	47.41	413.68	11.46	22.45	170.77	13.14
Chongming	6.49	40.31	401.72	10.03	23.00	161.89	14.21
Xujiahui	6.91	48.78	456.13	10.69	25.79	187.98	13.72
Nanhui	5.83	47.25	413.37	11.43	28.60	169.64	16.86
Jinshan	6.73	45.82	407.46	11.25	18.45	163.86	11.26
Qingpu	4.90	30.81	384.32	8.02	20.88	154.8	13.49
Songjiang	5.30	34.23	392.85	8.71	11.69	164.58	7.11
Fengxian	6.20	43.22	415.23	10.41	25.95	175.13	14.82

**Figure 5:**
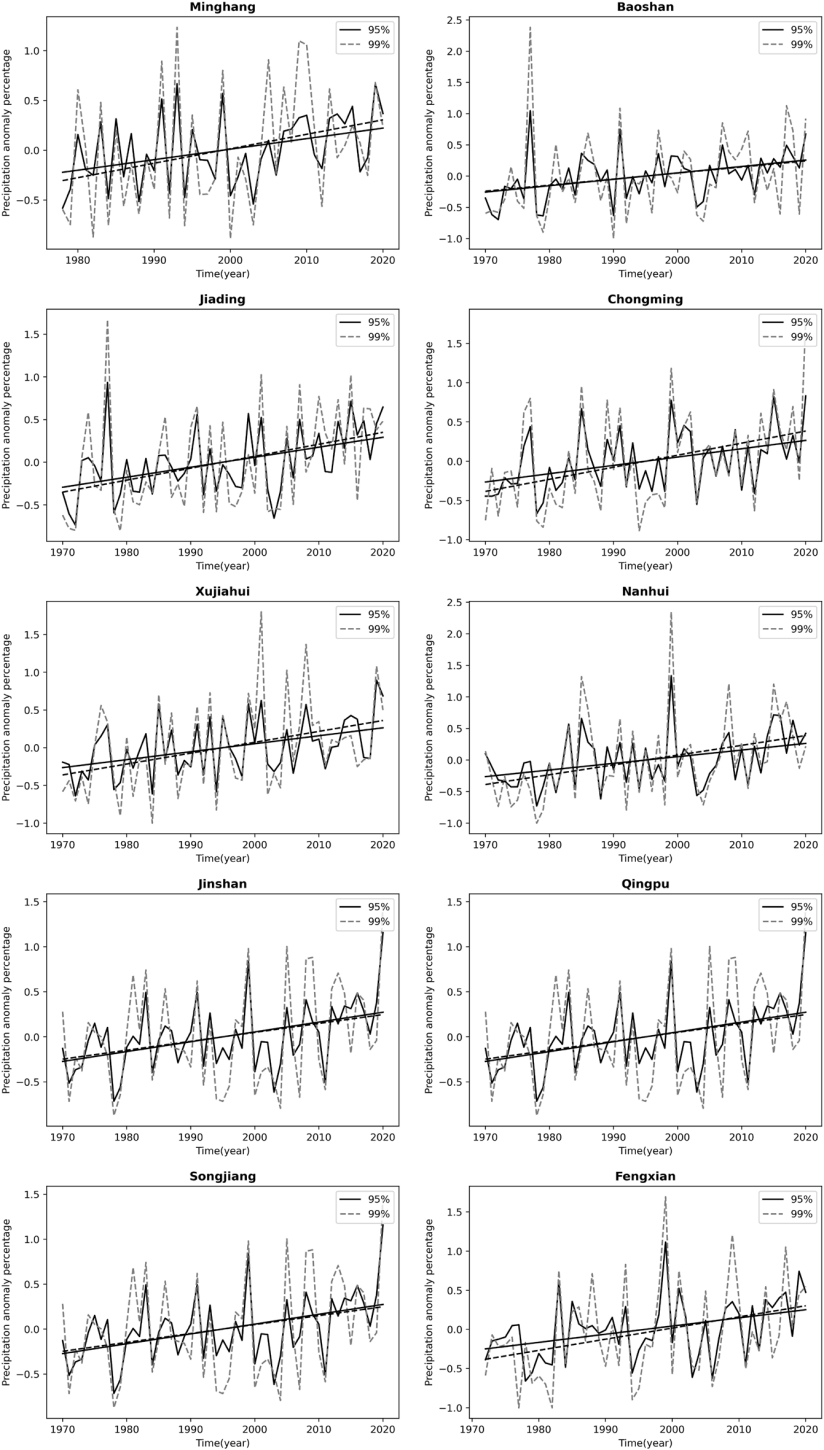
Trend of precipitation anomaly percentage.

We also used precipitation anomaly percentage (PAP) to reveal the changes of extreme precipitation. Figure 5 shows an increasing trend of PAP. There is a strong variability in extreme precipitation in this region, while the 99% threshold precipitation varies more.

**Figure 6:**
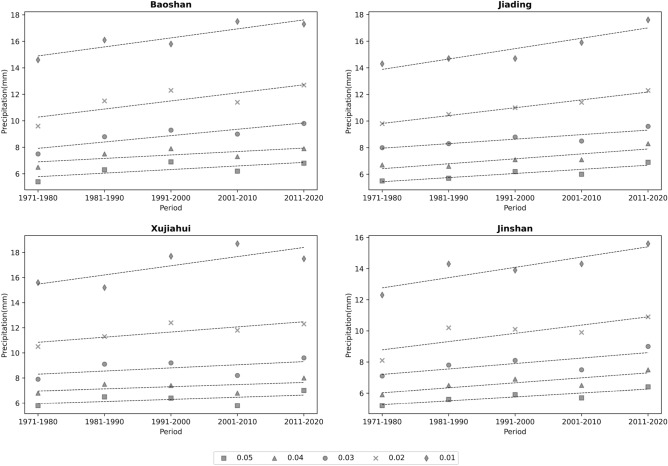
Variation of precipitation at different precipitation probabilities in each period.

To investigate the variability of the extreme portion of precipitation in the study area, the study period was divided into five stages: 1971–1980, 1981–1990, 1991–2000, 2001–2010, and 2011–2020. The cumulative probability of hourly precipitation (the cumulative probability of being greater than a certain precipitation value) within each phase was calculated, and the precipitation values corresponding to five sets of probabilities, 0.05, 0.04, 0.03, 0.02, and 0.01, were determined. We select 4 stations randomly (Baoshan, Jiading, Xujiahui, Jinshan) to show the variation of precipitation at each station with different precipitation probabilities. Figure 6 shows that the precipitation values corresponding to the five groups of probabilities at each station show an increase, and the precipitation values with smaller probabilities increase more significantly. It indicates that the extreme part of the precipitation probability distribution increases significantly. The above results regarding more significant growth rates of extreme precipitation and a significant increase in the extreme part of the precipitation probability distribution are broadly consistent with the current prevailing theory that the intensity of extreme precipitation increases with warming^41–45^.

### Urbanization impact

Land use obtained using satellite remote sensing data can visually reflect the urbanization process, so in most studies that examine the impact of urbanization on climate, researchers usually consider land use to characterize urbanization. Some researchers establish buffer zones of different distances with the station as the center of the circle, and express the urbanization rate by the area of urban land use within the buffer zone. For example, Huang et al.^35^ used urban land within a buffer zone of 5–30 km radius around the station to analyze the heat island effect of urban land expansion in Shanghai; He et al.^46^ used the land cover of a 1 × 1 km grid to analyze the relationship between urbanization rate and surface temperature; Tysa et al.^47^ established 20 buffer circles of 1–20 km to calculate the percentage of urban land use within the buffer circle and quantified the effect of urbanization on weather stations and adjacent areas. The results show that the correlation between urban land use and regional surface temperature trends within the 1–16 km buffer zone is highly significant. In order to study the influence of urbanization on extreme precipitation, a buffer zone with a moderate radius of 10 km was selected, and the mean value of urban land area within the buffer zone over the years was used to reflect the dynamic process of urbanization and to characterize the intensity of urbanization around the station.

**Figure 7:**
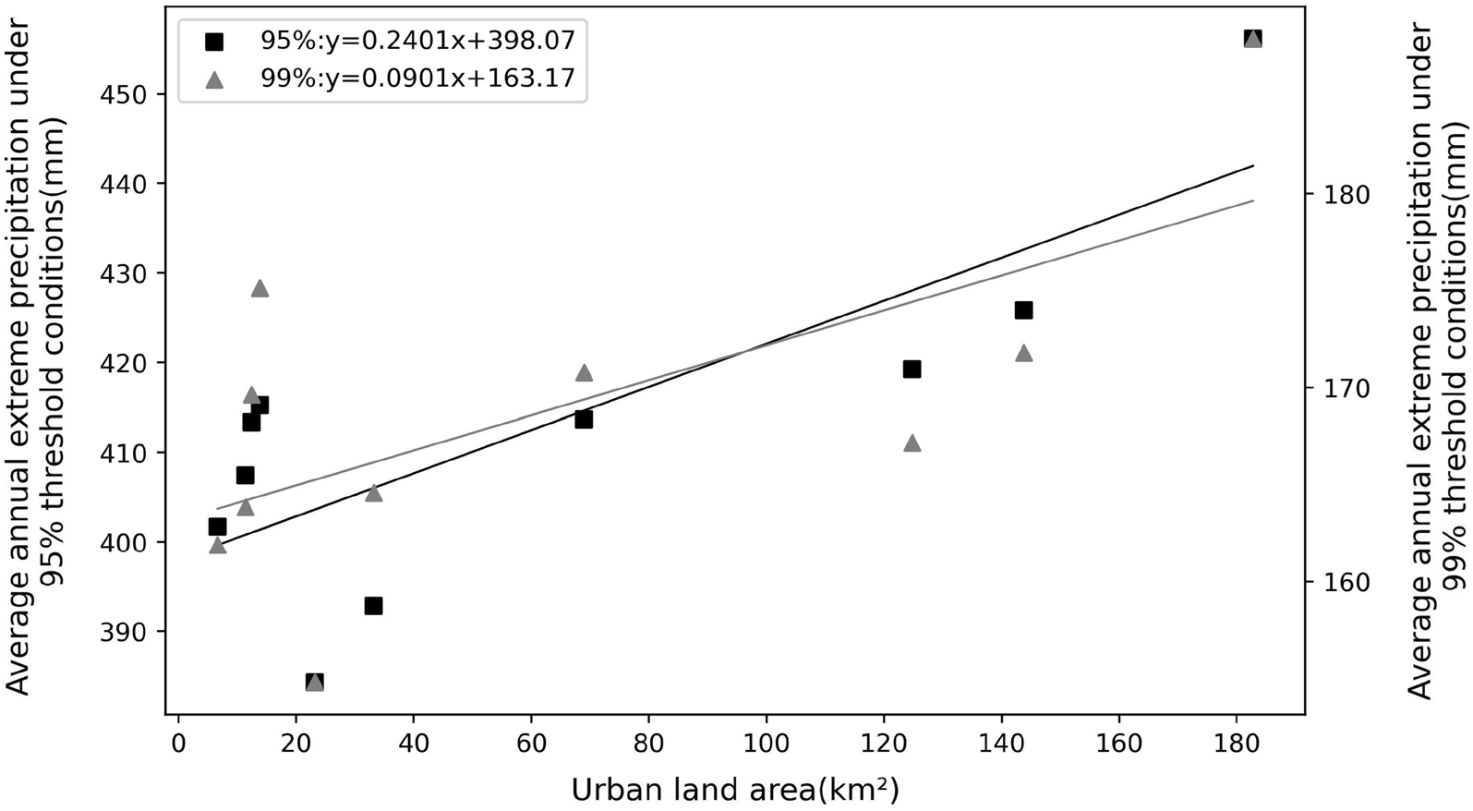
Plot of mean urbanization intensity versus average annual extreme precipitation for 5 periods.

We used the average urban land area of each site within 10 km for the five periods of 1980, 1990, 2000, 2010, and 2020 to characterize the average urbanization intensity of its area over the past 50 years (Table 6). The average annual extreme precipitation under 95% and 99% threshold conditions over 50 years were compared for each station (Table 7). Linear regression analysis and Pearson correlation analysis were done between the mean urbanization intensity within 10 km of each station and the average annual extreme precipitation (Fig. 7 and Table 8). The results of linear regression analysis illustrate that extreme precipitation shows a linear increase with the increase of urbanization intensity. Also, the Pearson correlation coefficients between the mean annual extreme precipitation and the mean urbanization intensity for the two threshold conditions are 0.797 (95% threshold) and 0.659 (99% threshold). They show a significant positive linear correlation. The average annual extreme precipitation increases by about 24.01 mm for each 100 km^2^ increase of urban area within 10 km around the site under 95% threshold condition and by about 9.01 mm under 99% threshold condition.

**Table 6:** The proportion of urban land area within 10 km of each station.

Year	Urban land area/km^2^									
	Minhang	Baoshan	Jiading	Chongming	Xujiahui	Nanhui	Jinshan	Qingpu	Songjiang	Fengxian
1980	27	52	18	2	125	2	0	6	6	3
1990	90	88	18	3	174	4	0	6	10	8
2000	131	127	39	5	194	6	0	10	18	10
2010	171	158	51	7	195	9	0	19	41	18
2020	300	199	219	16	226	41	57	75	91	30
Average of 5 periods	143.8	124.8	69	6.6	182.8	12.4	11.4	23.2	33.2	13.8

**Table 7:** Average urban land area and average annual extreme precipitation for 5 periods within 10 km of each station.

Station name	The average urban land area of 5 periods/km^2^	Average annual extreme precipitation/mm	
		95% threshold	99% threshold
Minhang	143.8	425.84	171.79
Baoshan	124.8	419.27	167.18
Jiading	69	413.68	170.77
Chongming	6.6	401.72	161.89
Xujiahui	182.8	456.13	187.98
Nanhui	12.4	413.37	169.64
Jinshan	11.4	407.46	163.86
Qingpu	23.2	384.32	154.8
Songjiang	33.2	392.85	164.58
Fengxian	13.8	415.23	175.13

**Table 8:** Pearson correlation analysis of mean urbanization intensity versus average annual extreme precipitation for 5 periods.

		Average annual extreme precipitation under threshold conditions(mm)	
		95%	99%
Urban land area(km^2^)	Pearson	.797**	.659*
	Significance	.006	.038
	Number	10	10

‘‘**’’indicates significant correlation with 0.01 significant level; ‘‘*’’ indicates significant correlation with 0.05 significant level; No labeling indicates that the correlation is not significant.

**Figure 8:**
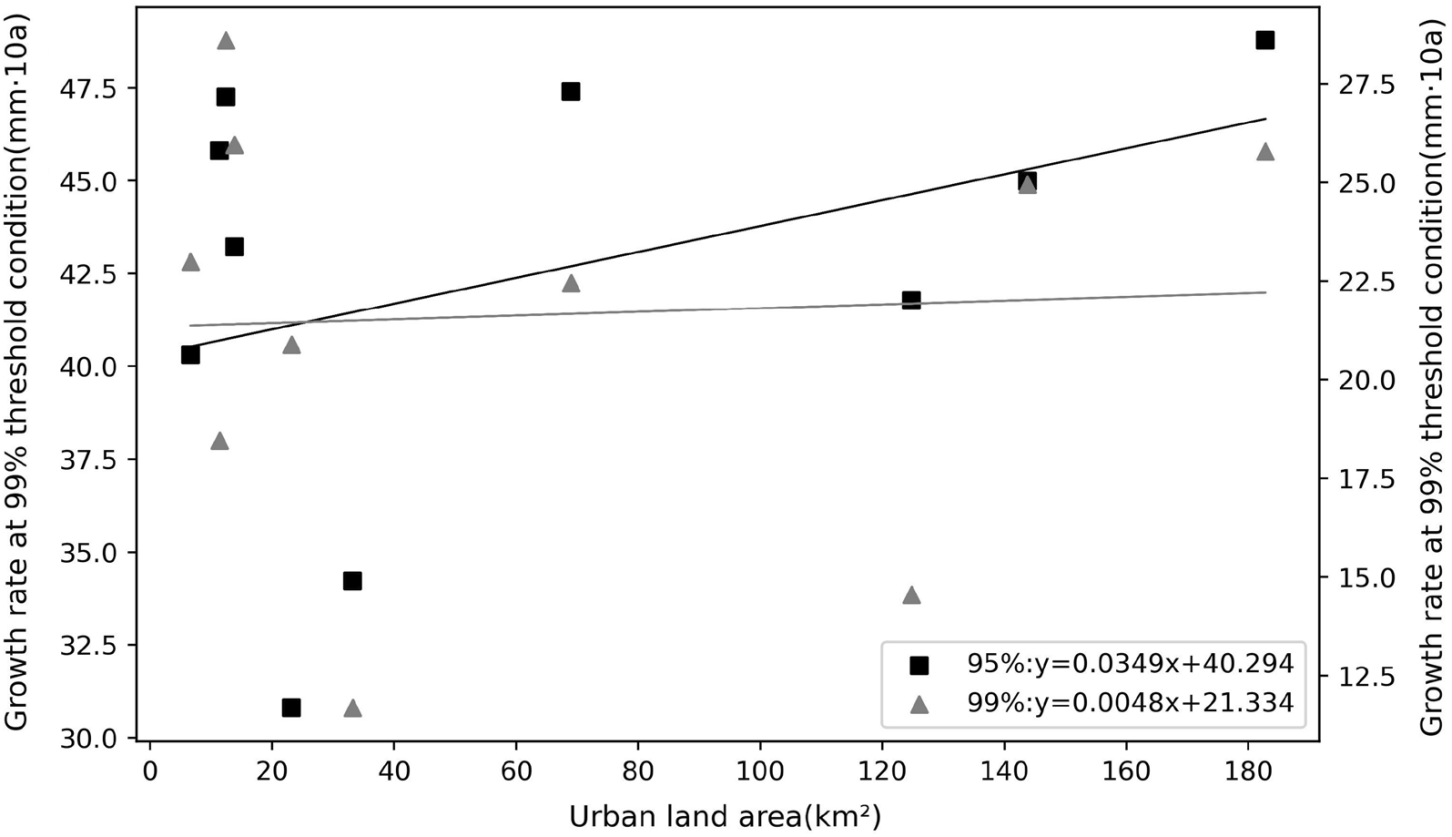
Average urbanization intensity versus the absolute rate of increase in extreme precipitation over 5 periods.

The previous analysis shows that the overall trend of extreme precipitation in Shanghai is increasing in the context of climate change. To investigate the influence of urbanization level on the degree of increase of extreme precipitation in the context of the overall increase of extreme precipitation, we further analyzed the relationship between urbanization intensity and the rate of increase of extreme precipitation. Figure 8 and Table 9 show that the correlation coefficient between urbanization intensity and the rate of increase of extreme precipitation is small and not significantly correlated. As can be seen, the intensity of urbanization has little effect on the growth rate of extreme precipitation.

**Table 9:** Pearson correlation analysis of mean urbanization intensity versus average annual extreme precipitation for 5 periods.

		Growth rate at threshold condition(mm·10a)	
		95%	99%
Urban land area(km^2^)	Pearson	.384	.058
	Significance	.274	.873
	Number	10	10

‘‘**’’indicates significant correlation with 0.01 significant level; ‘‘*’’ indicates significant correlation with 0.05 significant level; No labeling indicates that the correlation is not significant.

## Discussions

The differences in extreme precipitation across regions for the 95% and 99% thresholds (Table 3) are shown to be greater across regions for more extreme precipitation. This is because at the small regional scale of Shanghai, where the overall climatic background is consistent and topographic differences are not significant, the more conventional precipitation is necessarily more similar across regions, and the more extreme episodic precipitation is more irregular, hence the regional differences are greater. Both precipitation and extreme precipitation in Shanghai have shown an increasing trend in the last 50 years. The growth rate of extreme precipitation is higher than the average precipitation growth rate, and the growth rate of extreme precipitation under the 99% threshold is faster than that under the 95% threshold (Fig. 4 and Table 5). The more extreme precipitation increases more rapidly, which is consistent with the increasing frequency of extreme precipitation events in the context of global warming in recent years. Most studies on the trend of extreme precipitation only deal with the increasing/decreasing trend of extreme precipitation events^48–51^, without paying more attention to the difference in the rate of change of precipitation at different extremes. At the same time, extreme precipitation varies among stations in Shanghai, and we analyzed its relationship with urbanization intensity. The results show that the extreme precipitation is more in high urbanization areas. The enhanced effect of urbanization on extreme precipitation is consistent with the results of Song et al.^25^, Huang et al.^28^, Jiao et al.^52^ on extreme precipitation in cities such as Guangzhou and Beijing. The change in extreme precipitation in urban areas is actually a combined effect of multi-spatial-scale drives including global warming, sub-continental East Asian monsoon variability, regional climate change and urbanization, among which urbanization effect is very important^53^. Urbanization alters the thermal and dynamic characteristics of the land surface, affecting evaporation, changing atmospheric stability and turbulence, increasing precipitation in cities and their downwind directions (moderate confidence); while reducing bioaerosols but increasing anthropogenic aerosol emissions, affecting cloud physics and precipitation processes^54^. For example, the urban "heat island" effect accelerate water vapor transport and convection over the city and concentrate in the urban center or heat island center, while the increase in temperature also enhances the water retention capacity of the atmosphere, leading to the occurrence of extreme urban rainstorms. These effects are interrelated and together affect the precipitation characteristics of urban areas, highlighting the complexity and regional differences of urban precipitation mechanisms^55, 56^. Considering that the sites studied in this paper belong to the same climatic region, therefore, it can be inferred that the overall trend of precipitation is mainly caused by climate changes, while the local differences in precipitation among different sites can be attributed to the local differences in topography, energy flux in the cities caused by different degrees of urbanization around these sites. Highly urbanized areas have more water vapor transport and convection, and therefore more extreme precipitation. Of course, its specific physical causes need more attribution analysis studies in the future.

Since precipitation data samples are relatively small, uncertainties may exist in results of this paper due to the spatiotemporal variation of precipitation. In the future, further studies with more data are needed.

## Conclusions

Using hourly precipitation data from 10 meteorological stations in Shanghai from 1971 to 2020, we analyzed the temporal variation characteristics of extreme precipitation in Shanghai and the influence of urbanization on extreme precipitation and obtained the following conclusions:

1. The precipitation threshold limit value for each site in Shanghai are 5.7~6.3 mm per hour under 95% conditions and 14.3~17.16 mm per hour under 99% conditions. The coefficients of variation for the two extreme conditions are 0.04 and 0.06, respectively. The more extreme precipitation shows more significant differences between stations.
2. The extreme precipitation has been increasing at all stations over the last 50 years. The growth rate of extreme precipitation is significantly higher than that of average precipitation, while the extreme part of the precipitation probability distribution increases considerably. The relative rate of increase of average precipitation ranges from 4.9 to 6.96%/10a, the rate of increase of 95% threshold extreme precipitation is 8.02 to 11.46%/10a, and the rate of increase of 99% threshold extreme precipitation is 7.11 to 16.86%/10a. And the results of the MK trend test showed a significant increase trend of extreme precipitation. The precipitation anomaly percentage also indicates that the extreme precipitation tends to occur more frequently, and there is a strong variability in extreme precipitation in this region, while the 99% threshold precipitation varies more.
3. The extreme precipitation in Shanghai is positively correlated with regional urbanization around the site. Urbanization has an increasing effect on regional extreme precipitation, with more extreme precipitation in highly urbanized areas. Specifically, for every 100km^2^ increase in the area of towns within 10 km around the site, the average extreme precipitation under the 95% threshold condition increases by about 24.01 mm, and they are significantly positively correlated, with a Pearson correlation coefficient of 0.797; the average extreme precipitation under the 99% threshold condition increases by about 9.01 mm, and they are significantly positively correlated, with a Pearson correlation coefficient of 0.659. To some extent, regions with higher urbanization intensity have greater extreme precipitation growth.

## Supplementary Information

Supplementary Information.

## Data Availability

The raw data of the current study are available in the National Meteorological Information Centre of the China Meteorological Administration (http://data.cma.cn/) and Resource and Environment Science and Data Center of the Chinese Academy of Sciences(https://www.resdc.cn/Default.aspx).
