# Supplementary material for: Variation characteristics and the impact of urbanization of extreme precipitation in Shanghai
Source: Sci Rep. 2022 Oct 21;12:17618. doi: 10.1038/s41598-022-22352-4 (PMC9586963; doi:10.1038/s41598-022-22352-4)
Supplement: Supplementary file 1 — Supplementary Information. [file 41598_2022_22352_MOESM1_ESM.docx]

All 10 stations in Shanghai had been relocated^1^ (Table 1). In order to prevent the use of a test may cause omissions, we examined the homogeneity of the mean hourly precipitation at each station using the commonly used sliding t-test and Mann-Kendall test ^2,3,4^. The results are shown in Fig.1 and Fig.2.

Table 1 Stations information

| Station number | Station Name | Time Series | Relocation time^1^ |
| --- | --- | --- | --- |
| 58361 | Minhang | 1978-2020 | 19931101 |
| 58362 | Baoshan | 1971-2020 | 20030101 |
| 58365 | Jiading | 1971-2020 | 19990813 |
| 58366 | Chongming | 1971-2020 | 20030501 |
| 58367 | Xujiahui | 1971-2020 | 19990701 |
| 58369 | Nanhui | 1971-2020 | 20011101 |
| 58460 | Jinshan | 1971-2020 | 20030801 |
| 58461 | Qingpu | 1971-2020 | 19990101 |
| 58462 | Songjiang | 1971-2020 | 20030801 |
| 58463 | Fengxian | 1972-2020 | 19970101;20100101 |

| 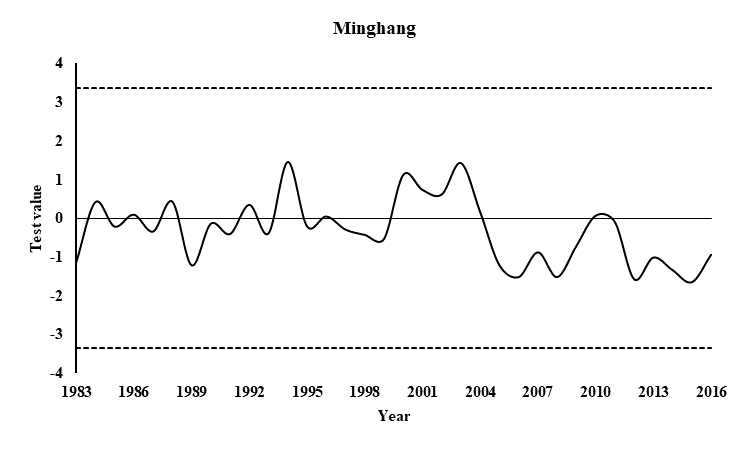 | 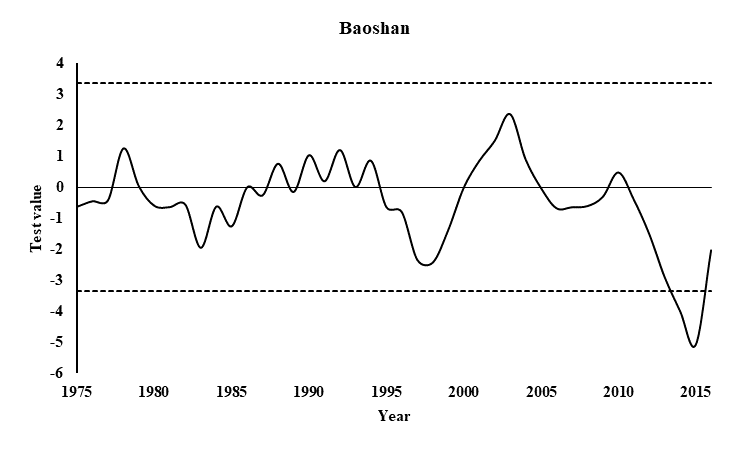 |
| --- | --- |
| 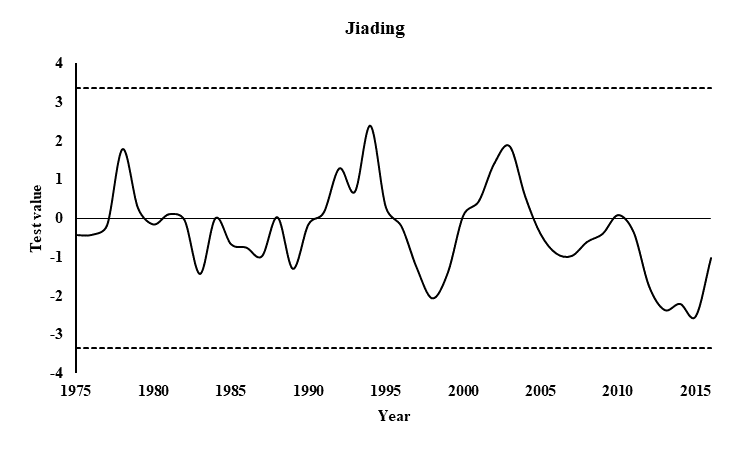 | 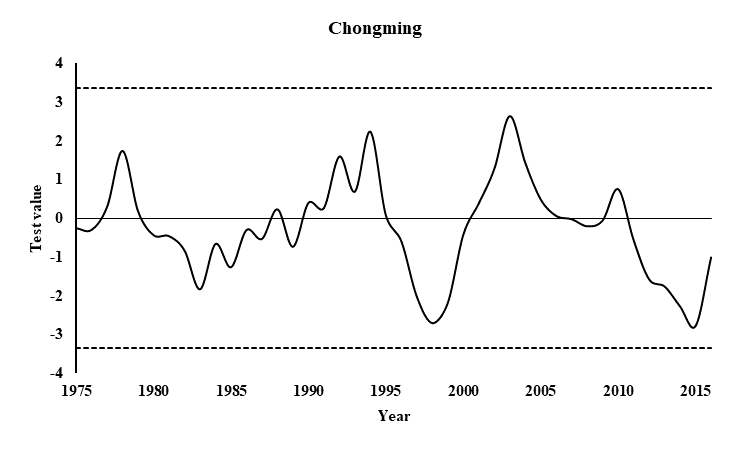 |
| 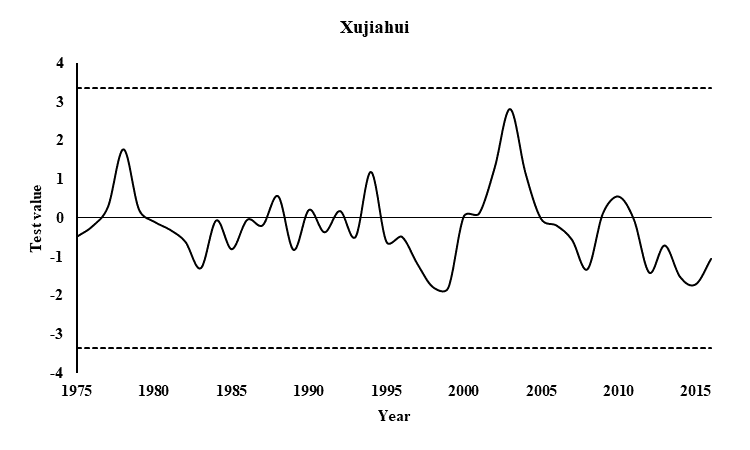 | 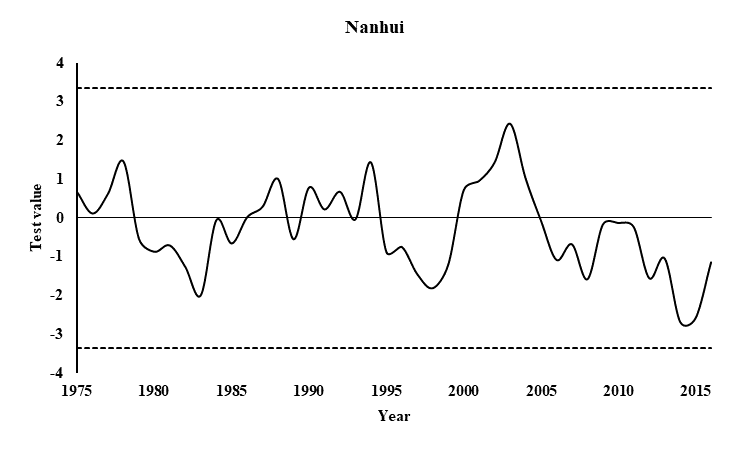 |
| 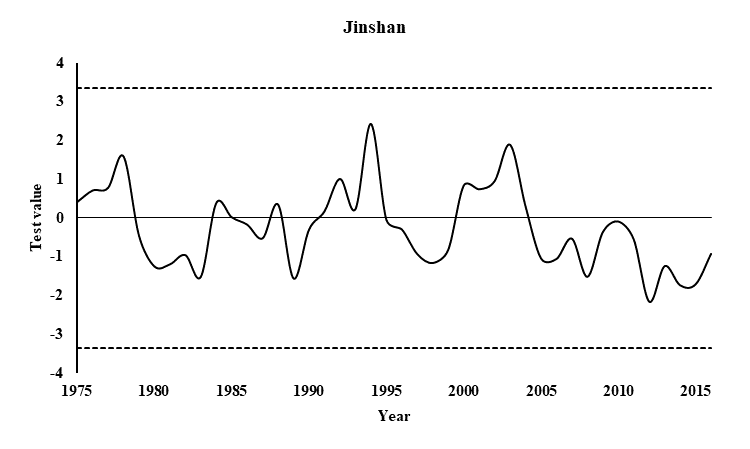 | 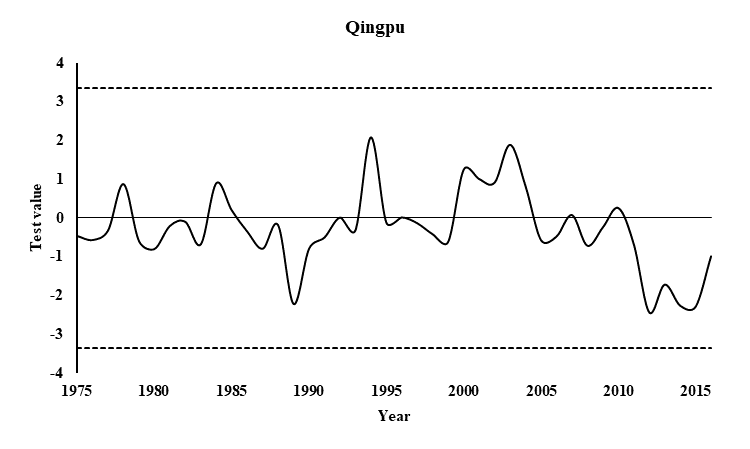 |
| 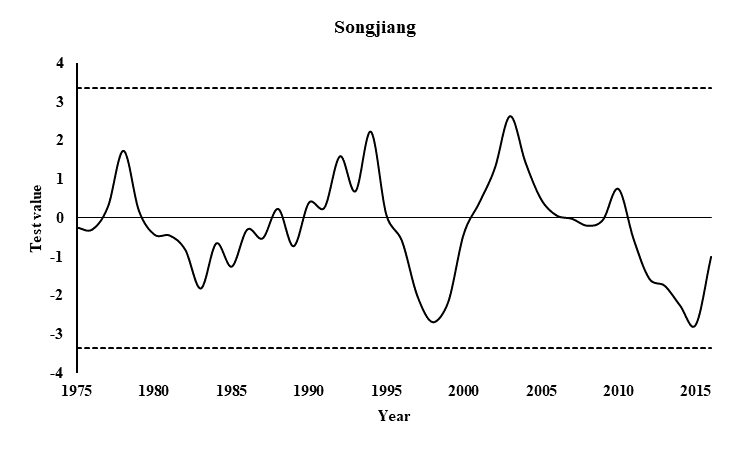 | 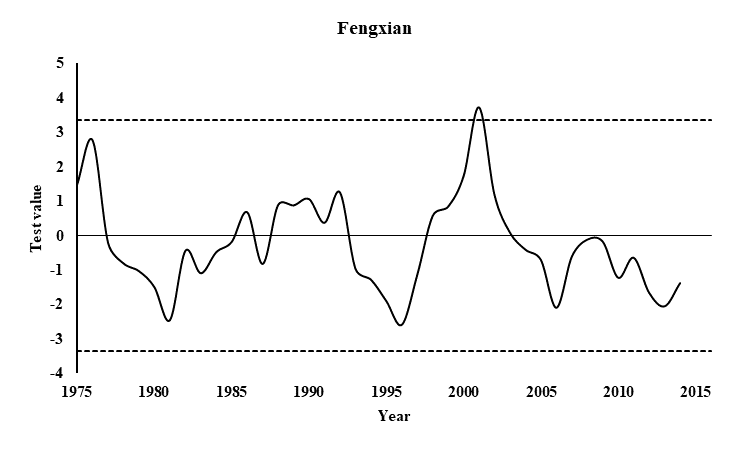 |
| Fig.1 Sliding t-test for long series mean hourly precipitation (thick solid lines are t-test values; dashed lines are 0.01 significance levels) | |

| 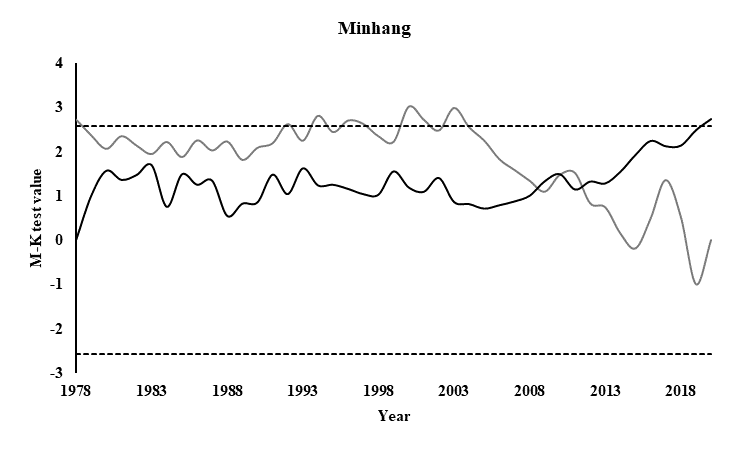 | 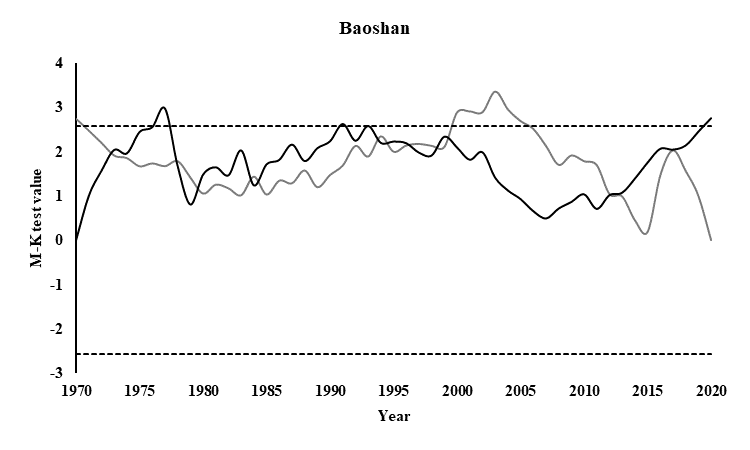 |
| --- | --- |
| 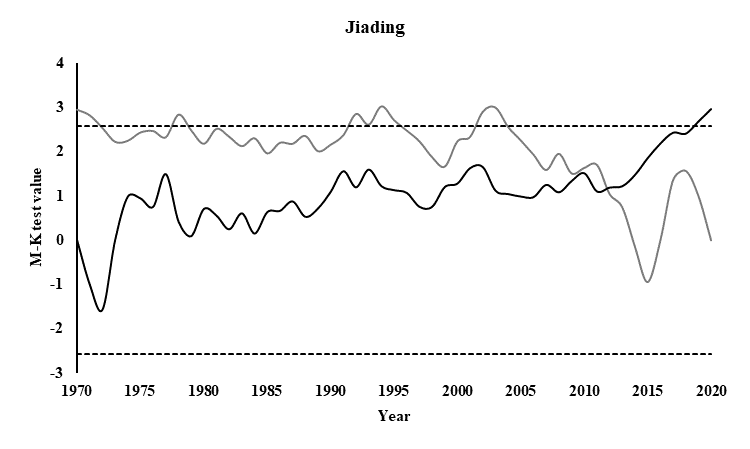 | 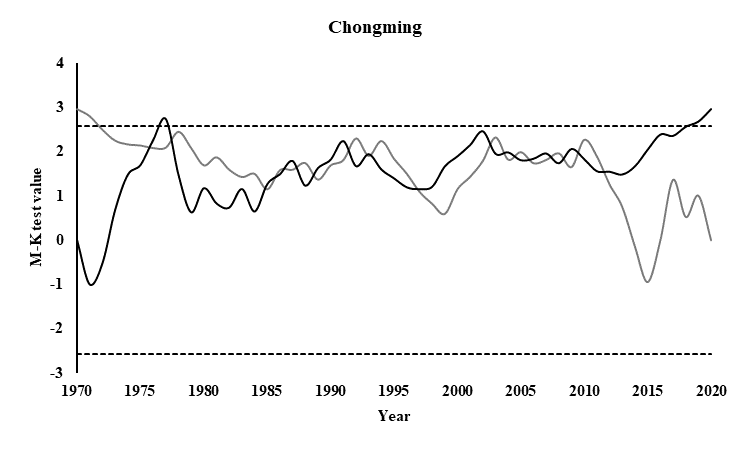 |
| 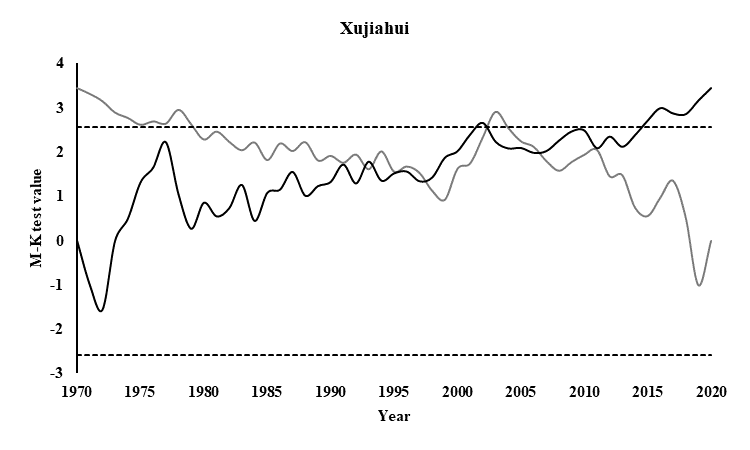 | 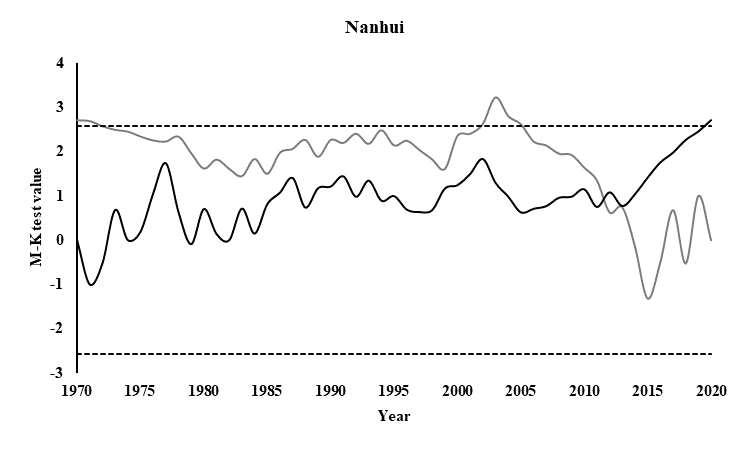 |
| 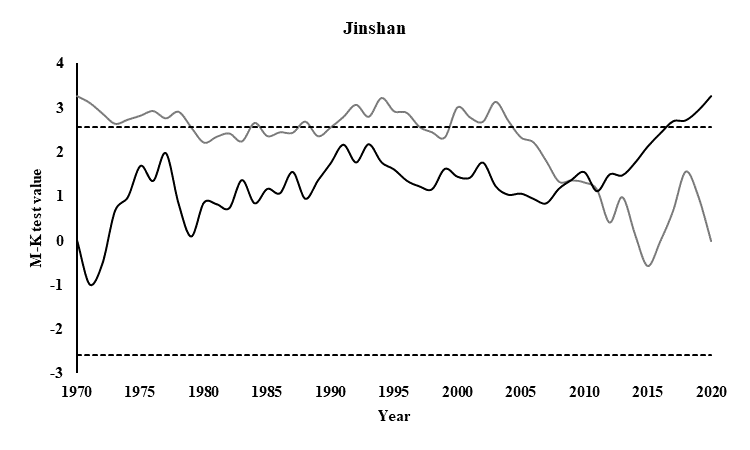 | 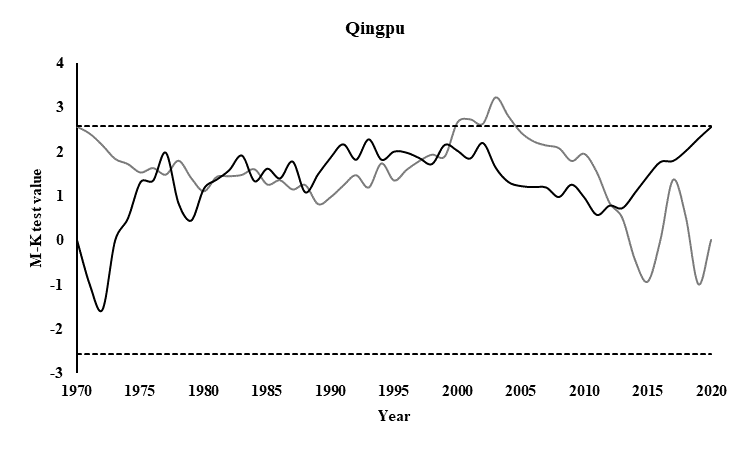 |
| 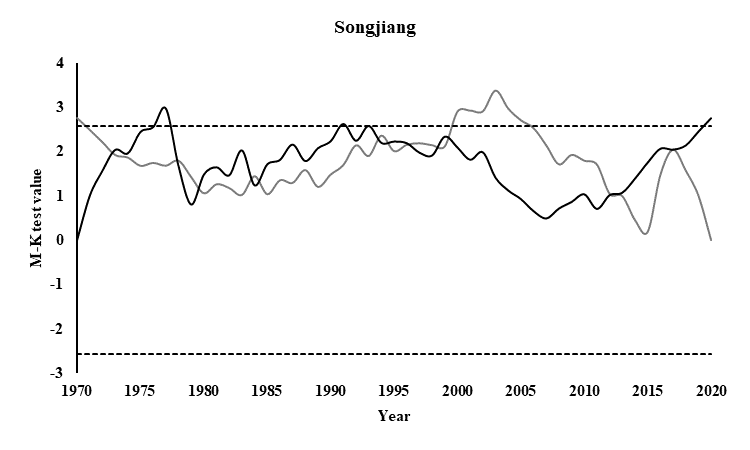 | 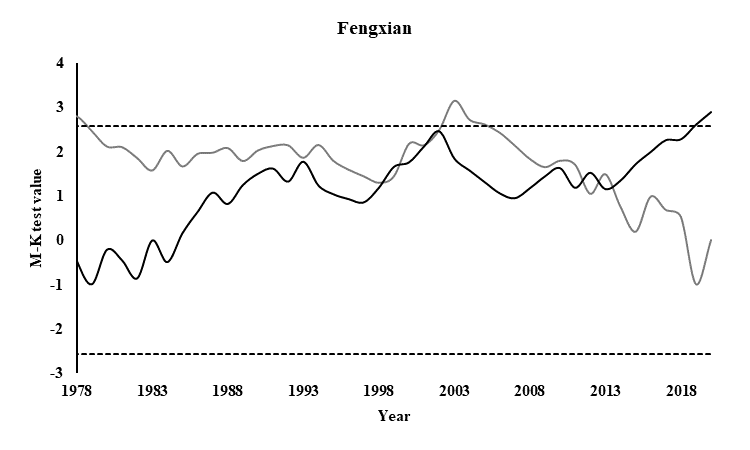 |
| Fig.2 Mann-Kendall test for long series mean hourly precipitation (The lighter colored solid line is the UB statistic; the darker colored solid line is the UF statistic; dashed lines are 0.01 significance levels) | |

As Fig.1, there was no abrupt change in precipitation series before and after the relocation time for 8 stations, so it was concluded that the relocation did not affect the homogeneity of the data. In particular, although abrupt changes occurred in 2015 and 2011 at Baoshan and Fengxian stations, no abrupt changes occurred around the time of relocation (2003 and 1999, 2010). Fig.2 shows that the intersection of UF and UB is present except for the Xujiahui site, which indicates the presence of abrupt changes points, but does not break the critical value of 0.01 significance level. Therefore, it can be assumed that no significant abrupt changes occurred. The significant abrupt change at the Xujiahui site occurred in 2003, while the abrupt change occurred in 1999 when the site was relocated, indicating that the relocation did not have an abrupt effect on the precipitation sequence. The results of the combined sliding t-test and MK test, the possible years of abrupt changes in the evaluated hourly precipitation at each site are shown in Table 2. In summary, the relocation of the stations in Shanghai does not have a significant abrupt effect on the long series of precipitation, it can be assumed that the relocation did not change the homogeneity of the data, and the data used in this paper are reliable.

| Station number | Station Name | Relocation time^1^ | Year of abrupt change |
| --- | --- | --- | --- |
| 58361 | Minhang | 19931101 | - |
| 58362 | Baoshan | 20030101 | 2015 |
| 58365 | Jiading | 19990813 | - |
| 58366 | Chongming | 20030501 | - |
| 58367 | Xujiahui | 19990701 | 2003 |
| 58369 | Nanhui | 20011101 | - |
| 58460 | Jinshan | 20030801 | - |
| 58461 | Qingpu | 19990101 | - |
| 58462 | Songjiang | 20030801 | - |
| 58463 | Fengxian | 19970101;20100101 | 2001 |

**Reference**

1. Huang. W. Quantifying the effect of urbanization on urban heat island and its climatological analysis using satellite and ground-based data. *Zhejiang University* (2018).
2. Wei, F. Modern climate statistical diagnosis and prediction technology. *China Meteorological Press* (2007)*.*
3. Xu, Q., Gao Q., Hu, J., & Hu, X. Homogeneity Test and Analysis of Meteorological Data before and after Qianjiang Station Moving. *Plateau Meteorology*. **30(06)**:1709-1715(2011).
4. Meng, R., Yang, L., Tian, Y., & Meng, K. Comparative analysis and homogeneity test of climate data before and after of Foping Station Moving. *Journal of Anhui Agri. Sci*. **41(21)**:9019-9022(2013).
